# A Randomized Controlled Trial Comparing Alloderm-RTU with DermACELL in Immediate Subpectoral Implant-Based Breast Reconstruction

**DOI:** 10.3390/curroncol28010020

**Published:** 2020-12-25

**Authors:** Angel Arnaout, Jing Zhang, Simon Frank, Moein Momtazi, Erin Cordeiro, Amanda Roberts, Ammara Ghumman, Dean Fergusson, Carol Stober, Gregory Pond, Ahwon Jeong, Lisa Vandermeer, Brian Hutton, Mark Clemons, on behalf of the REaCT Investigators

**Affiliations:** 1 Department of Surgery, The Ottawa Hospital and University of Ottawa, Ottawa, ON K1Y 4E9, Canada; anarnaout@toh.ca (A.A.); jzhang1@toh.ca (J.Z.); sfrank@toh.ca (S.F.); mmomtazi@toh.ca (M.M.); ecordeiro@toh.ca (E.C.); amanda.roberts@sunnybrook.ca (A.R.); ammara.ghumman@gmail.com (A.G.); 2 Ottawa Hospital Research Institute and University of Ottawa, Ottawa, ON K1H 8L6, Canada; dafergusson@ohri.ca (D.F.); cstober@ohri.ca (C.S.); ahwonjeong023@gmail.com (A.J.); lvandermeer@ohri.ca (L.V.); bhutton@ohri.ca (B.H.); 3 Clinical Epidemiology Program, The Ottawa Hospital Research Institute, Ottawa, ON K1H 8L6, Canada; 4 Juravinski Cancer Center, McMaster University, Hamilton, ON L8S 4L8, Canada; gpond@mcmaster.ca; 5 Department of Medicine, Division of Medical Oncology, The Ottawa Hospital and University of Ottawa, Ottawa, ON K1H 8L6, Canada

**Keywords:** acellular dermal matrix, mastectomy, immediate breast reconstruction

## Abstract

Background: The effectiveness of different acellular dermal matrices (ADM) used for implant-based reconstruction immediately following mastectomy is an important clinical question. A prospective randomized clinical trial was performed to evaluate the superiority of DermACELL over Alloderm-RTU in reducing drain duration. Methods: Patients undergoing mastectomy with subpectoral immediate and permanent implant-based breast reconstruction were randomized to Alloderm-RTU or DermACELL. The primary outcome was seroma formation, measured by the duration of postoperative drain placement. Secondary outcomes included: post drain removal seroma aspiration, infection, redbreast syndrome, wound dehiscence, loss of the implant, and unplanned return to the operating room. Results: 62 patients were randomized for 81 mastectomies (41 Alloderm-RTU, 40 DermACELL). Baseline characteristics were similar. There was no statistically significant difference in mean drain duration (*p* = 0.16), with a trend towards longer duration in the Alloderm-RTU group (1.6 days; 95%CI, 0.7 to 3.9). The overall rate of minor and major complications were statistically similar between the two groups; although patients with Alloderm-RTU had 3 times as many infections requiring antibiotics (7.9% vs. 2.5%) with a risk difference of 5.4 (95%CI −4.5 to 15.2), and twice as many unplanned returns to the operating room (15.8% vs. 7.5%) with a risk difference of 8.3 (95% CI −5.9 to 22.5) as DermACELL. Conclusion: This is the first prospective randomized clinical trial comparing the two most commonly used human-derived ADMs. There was no statistically significant difference in drain duration, minor, or major complications between DermACELL over Alloderm-RTU in immediate subpectoral permanent implant-based breast reconstruction post-mastectomy.

## 1. Introduction

Breast reconstruction following mastectomy can be accomplished using the patient’s own tissues (autologous) or implantable prosthetic devices such as implants and tissue expanders (alloplastic reconstruction) [1,2]. Acellular dermal matrix (ADM) typically of human, bovine, or porcine origin are increasingly being used for lower pole coverage in immediate implant-based reconstruction [3]. However, evidence for the clinical safety of ADM use in implant-based breast reconstruction is sparse, with reported complication rates from 4.0 to 50.0 percent [4,5,6,7]. As such, The Association of Breast Surgery and the British Association of Plastic, Reconstructive and Aesthetic Surgeons provided joint guidelines in 2013 for use of acellular dermal matrices in breast reconstruction [8,9]. There were three key criteria for maintaining quality and safety: (1) complications leading to implant loss should occur in less than 5% of patients, (2) fewer than 5% of patients should require a return to the operating room for correction of local complications within 30 days of the index operation, and (3) fewer than 10% of patients should require antibiotics within 3 months of surgery for suspected infection.

Two of the most commonly used human-derived ADM products in North America are Alloderm Ready-To-Use (RTU; Allergan Inc., Madison, NJ, USA) and DermACELL (Stryker Corp., Kalamazoo, MI, USA) [3,10,11,12]. In current practice, the selection of ADM is usually based on surgeon preference, hospital budgets and negotiated vendor contractual agreements. Despite differences in their level of sterility, consistency and thickness of the biologic material as well as cost, we are unaware of any prospective randomized trials comparing them directly [9,13,14].

We proposed a study comparing the two commonly used ADMs in a population of patients undergoing immediate reconstruction with a permanent implant at the time of their mastectomy, using the REaCT prospective pragmatic clinical trial methodology for comparing standard of care interventions [15,16]. This study aims to evaluate postsurgical outcomes with the use of Alloderm-RTU compared to DermACELL in immediate subpectoral implant-based breast reconstruction. The primary outcome is seroma formation, as prolonged seroma is the most common complication following implant-based breast reconstruction using ADMs [17,18,19,20]. Prolonged seroma formation can progress to infection and prosthesis loss [20,21]. It may also interfere with the process of integration and thereby the success of the reconstruction [13,20,21]. In addition, time to drain removal, while often overlooked by surgeons, is a common complaint about patients, as it can result in postoperative discomfort or pain and increased office visits [20,21].

## 2. Methods

### 2.1. Study Design and Population

This was a single centre, open-label, prospective randomized trial. Patients undergoing mastectomy and immediate subpectoral permanent implant-based breast reconstruction at the Ottawa Hospital, Ottawa, Canada were approached for potential study eligibility. Eligibility criteria included: female patients between the ages of 20 and 80, with a planned subpectoral immediate reconstruction at the time of a unilateral or bilateral, therapeutic or prophylactic, skin or nipple-sparing mastectomy, and ability to provide verbal consent. Exclusion criteria included patients undergoing tissue expander insertion or prepectoral implant-based reconstruction at the time of the mastectomy. The study was approved by the Ottawa Health Science Network Research Ethics Board (Protocol 20160568-01H, approved 14 August 2016). The trial was preregistered on clinicaltrials.gov (NCT03064893).

### 2.2. The REthinking Clinical Trials (REaCT) Program and Integrated Consent Process

The REaCT methodology has been used in systemic therapy, imaging and interventional studies [15,16], however, this is the first REaCT trial to evaluate two standards of care surgical interventions. Potentially eligible patients were informed by their breast or plastic surgeon about the risks and benefits of the two different standards of care ADMs. This integrated consent model is akin to a typical conversation between the physician and the patient. The physician gave the patient a consent template that briefly outlines the study. After the patient’s questions were answered and the patient was willing to enter the study, this clinical interaction was documented in the patient’s electronic medical record (EMR). There was no written consent form and a clinical research associate (CRA) did not perform the consent process.

### 2.3. Randomization

Eligible and consenting patients were randomized by the CRA using a web-based program developed by the Ottawa Methods Centre. Patients were randomized 1:1 to either Alloderm-RTU or DermACELL using a permuted variable block design with block sizes of 4 and 6, with no stratification factors. The randomization was at the patient level. Patients who were undergoing bilateral mastectomy and bilateral immediate implant reconstruction were randomized to the same type of ADM for each breast, and data on each breast collected. The plastic surgeon was informed of the randomization arm on the day of surgery, immediately before the procedure and both Alloderm-RTU and DermACELL were available in the operating room. All the breast oncologic (AA, EC, AR) and reconstructive (JZ, SF, MM) surgeons at the institution were involved in the study. The oncologic surgeons performed nipple or skin-sparing mastectomies; while the plastic surgeons performed the immediate implant reconstruction.

### 2.4. Surgical Technique and Drain Management

The implant pocket was defined with the elevation of pectoralis major muscle and the ADM (unfenestrated) was anchored with absorbable sutures to the inframammary fold and the inferior part of the lateral boundary along the anterior maxillary line. Triple antibiotic solution (Bacitracin, Cefazolin/Gentamicin) was used to irrigate both the pocket and implant. The implant was placed under the elevated pectoralis major muscle and the free muscle edge secured to the ADM. Two drains were placed; one in the subpectoral and one in the subcutaneous pocket. The mastectomy skin flaps were closed over the pectoralis muscle and ADM in the standard fashion. The techniques used in reconstructing breasts did not differ between products other than the specific manufacturer’s instructions for each individual ADM and the surgeons all used the same technique. Alloderm-RTU was prepared using a minimum 2-min soak in either sterile saline or lactated Ringer solution for a total of two times. DermACELL was ready for use from the package. Plastic surgeons used their own discretion with regard to the size of the ADM used. In patients who had bilateral reconstruction, the same ADM was used for each breast. The decision for mastectomy incision and nipple preservation was made jointly by the oncologic and plastic surgeon. During the primary operation, two closed suction drains, Jackson Pratt, French gauge 14, were used for each breast. The drains were kept in place until the output was less than 20 mL per 24 h, for a maximum of 14 days. All patients and/or caregivers were taught on the daily measurement of drain outputs and drain output records were reviewed by an outpatient homecare nurse (arranged for every patient) or surgeon prior to draining removal. In addition to the usual IV antibiotic prophylactic dose upon induction of the general anesthetic, patients also took oral antibiotics for one week postoperatively.

### 2.5. Data Collection

Baseline demographic, clinical characteristics and endpoint data were collected from the patient’s medical record. The routine schedule of postoperative follow-up of these patients was 2 weeks and 6 months for the breast surgeon and 1 week, 2 weeks, and 6 months for the plastic surgeon. Patients having bilateral mastectomy had each breast evaluated separately for primary and secondary outcomes.

### 2.6. Outcomes

#### 2.6.1. Primary Outcomes

The primary outcome was the duration of postoperative drain placement, as a surrogate endpoint for the extent of seroma formation. If the patient had multiple drains, the date of removal of the last drain was used.

#### 2.6.2. Secondary Outcomes

Secondary outcomes included: episodes of seroma aspiration following drain removal, removal of the implant, unplanned revisional surgery/return to the operating room, wound infection requiring antibiotics, wound dehiscence or need for debridement, capsular contracture (as identified by the plastic surgeon), and red breast syndrome. Red breast syndrome was defined as erythema occurring directly over the ADM. Revisional surgeries for oncological reasons (excision of positive margins, removal of the implant prior to adjuvant radiation) and aesthetic reasons (patient choice, nipple reconstruction) were not included. The number of total postoperative clinic visits with the plastic surgeon (beyond the routine) was also compared. All outcomes were measured within 6 months of the initial surgery.

### 2.7. Sample Size and Statistical Analysis

The primary outcome of the study was drain duration. The literature reports the mean drain duration for subpectoral immediate prosthetic based reconstruction with ADM to be in the range of 8–12 days [19,21,22,23]. As such, the plastic and breast surgeons in this study determined that a minimum of 4 days in drain duration between the two arms was considered clinically significant. A two-sample, two-sided, α = 0.05, *t*-test would achieve 80% power with a minimum of 52 patients, assuming a common standard deviation of 5. Allowing for a 10% study drop-out rate, we aimed to enroll a total of 58 patients or 29 patients per arm.

All data were analyzed with SPSS software (version 20, IBM, Armonk, NY, USA). Descriptive statistics were used to summarize patient characteristics, treatment information and outcomes overall, and by treatment arm. The Wilcoxon rank-sum test and, Fisher’s exact test were used to compare intervention arms for continuous and dichotomous variables, respectively. As some outcomes were measured using each breast as the unit of analysis, supportive analyses were performed using Generalized estimating equations (GEEs) which account for the correlation between breasts from the same patient. The difference in the mean and difference in proportions were calculated along with 95% confidence intervals using the Satterthwaite (continuous) and Wald (categorical) methods. All tests were two-sided, and a *p*-value of 0.05 or less was considered statistically significant.

## 3. Results

### 3.1. Patient Characteristics

**Figure 1:**
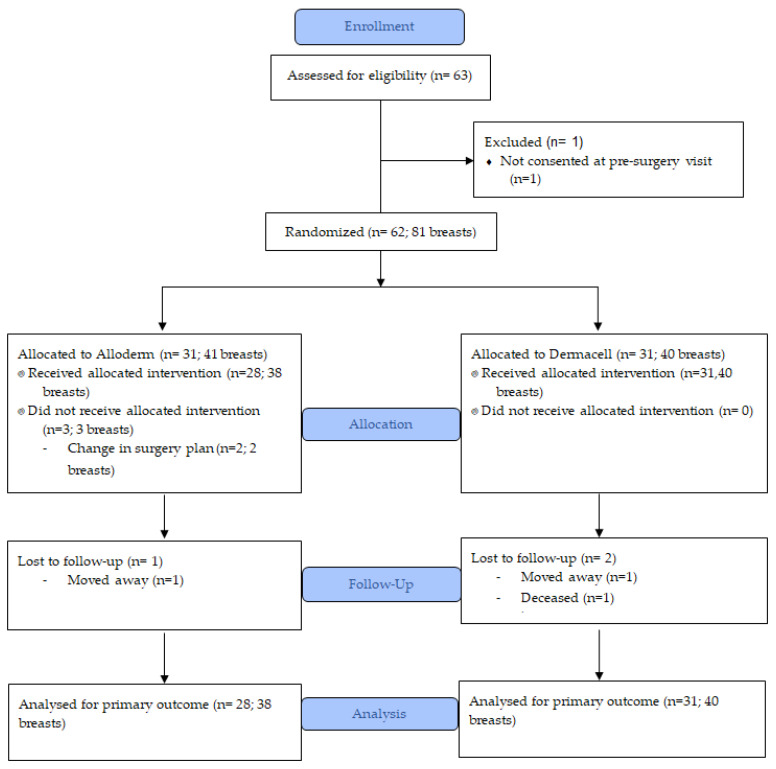
CONSORT diagram.

Between June 2016 and October 2018, 63 patients were approached, and 62 agreed to randomization. The number of patients randomized to the Alloderm-RTU and DermACELL arms were 31 (50%) and 31 (50%), respectively. The consort diagram is shown in Figure 1. The baseline characteristics (*n* = 62 patients, 81 breasts) are shown in Table 1. The mean age was 47.8 years (SD 11.1) for Alloderm-RTU and 51.4 years (SD 11.0) for DermACELL. Mean body-mass index was similar in both groups with 24.9 kg/m^2^ (SD 4.6) for Alloderm-RTU and 24.9 kg/m^2^ (SD 4.9) for DermACELL. Five patients (16.1%) in Alloderm-RTU and three patients (9.7%) in the DermACELL group had heart disease. Other baseline characteristics were similar in both groups included smoking history, diabetes, heart disease, and preoperative chemotherapy. Interestingly, although the proportion of patients with a D cup or higher breast size, grade III ptosis or greater, and radiation were higher in the Dermacell group, there was no statistically significant difference between the two groups. Three patients did not receive the allocated ADM and did not have outcome data: two patients had a change of reconstruction plan which did not require ADM and one surgeon accidentally used the wrong type of ADM. In total, 59 patients (78 breasts; 38 Alloderm-RTU and 40 DermACELL) who underwent ADM-assisted breast reconstruction were included in the final analysis. Nineteen patients had bilateral mastectomy and reconstruction.

### 3.2. Surgical Characteristics

Among the 78 mastectomies, there were 25 prophylactic and 53 therapeutic mastectomies (Table 2). Nipple-sparing mastectomy was performed in 40 breasts (51.3%), and among these, 34 breasts had an incision at the inframammary fold. The weights of 26 out of 78 (33.3%) mastectomy specimens were not available in the pathology reports, which resulted in a higher but not statistically significant different median mastectomy weight (600 g (IQR 324 g to 714 g) for Alloderm-RTU versus 387 g (IQR 269 g to 542 g) for DermACELL). Indications for surgery, type of mastectomy, type of incision, and incidence of axillary surgery were similar between the two groups.

### 3.3. Primary Outcome Measure

The mean duration of drain placement was 10.8 days (standard deviation, SD, 5.5) with Alloderm-RTU and 9.2 days (SD 4.5) with DermACELL (Table 3). The risk difference in favour of DermACELL was 1.6 days (*p* = 0.16, 95%CI −0.7 to 3.9).

### 3.4. Secondary Outcomes Measures

Complications that occurred during the first 6 months after implant placement were included in the analyses (Table 3). Wound infections requiring antibiotics occurred in 3 breasts (7.9%) with Alloderm-RTU and 1 (2.5%) with DermACELL (*p* = 0.35), with a risk difference of 5.4 (95%CI −4.5 to 15.2). Unplanned reoperation due to complications was necessary for 6 breasts (15.8%) with Alloderm-RTU and 3 breasts (7.5%) with DermACELL (*p* = 0.30), with a risk difference of 8.3 (95%CI −5.9 to 22.5). Reasons for reoperation included removal of implant (*n* = 4), infection (*n* = 3), skin necrosis (*n* = 3), wound dehiscence (*n* = 2), hematoma (*n* = 1), and suture removal (*n* = 1). No patient required more than one reoperation.

Minor complications were defined as the occurrence of a seroma requiring aspiration, red breast syndrome, wound dehiscence, wound infection, hematoma, skin necrosis, and capsular contracture. The rate of minor complications in the Alloderm-RTU group was 36.8% (*n* = 14) and 32.5% (*n* = 13) in the DermACELL group. Using Poisson regression assuming no correlation between breasts, this was not statistically significant (*p* = 0.57). Major complications were defined as any complication requiring reoperation. The rate of major complications was 15.8% (*n* = 6) in the Alloderm-RTU group and 7.5% (*n* = 3) in the DermACELL group and was not statistically significant (*p* = 0.30).

### 3.5. Post-Surgical Treatment and Follow-Up

The median number of postoperative clinic visits with the plastic surgeon was 4 for Alloderm-RTU (IQR 3, 5) and 3 for DermACELL (IQR 3, 4.5) (risk difference 0.1, 95%CI −1.1 to 1.2, *p* = 0.13) (Table 4). Adjuvant chemotherapy was administered in 16 patients (27.1%; 9 (32.1%) in Alloderm-RTU and 7 (22.6%) in the DermACELL group. Adjuvant radiotherapy was administered in 13 breasts (16.7%); 7 (18.4%) in Alloderm-RTU and 7 (22.6%) in DermACELL.

## 4. Discussion

To help reduce practice variation as well as improve fiscal responsibility in healthcare, it will be increasingly important to perform studies comparing standard of care interventions. It is recognized that having breast restoration immediately post-mastectomy (i.e., direct to implant breast reconstruction) significantly improves patient care [24], wellbeing [25,26], and also offers cost savings from sparing a second surgery [27,28,29]. ADM use has also been associated with improved aesthetic outcomes [30,31], and thus an ADM-assisted breast reconstruction has become the preferred approach at many centres globally [32,33,34,35,36]. For USD $25–$35 per square cm (most commonly used sizes are 6 cm × 16 cm), there is a need to perform high-quality clinical trials evaluating the performance, safety, and post-surgical outcomes of the various ADMs in this era of value-based care [27,28,29].

This current trial is the first prospective randomized trial comparing the specific ADMs, Alloderm-RTU and DermACELL, two of the most commonly used ADMs in North America [3,9,10,37]. Our study is the first randomized clinical trial comparing the two products in a head-to-head fashion. The differences between the two products include (a) the level of sterility, with DermACELL being sterilized to a sterility assurance level of 10^−6^ while Alloderm-RTU is sterilized to 10^−3^; (b) the consistency and thickness of the biologic material; and (c) a potential difference in cost (product cost is based on individually negotiated vendor-hospital contracts). Currently, at our hospital, a 6 cm × 16 cm piece of Alloderm-RTU is more expensive than DermACELL by approximately 35%. Hospitals and surgeons may choose one ADM product over another based on the relative importance and value they place on each of these factors. For example, proponents of DermACELL advocate for its increased sterility; but whether the difference in sterility translates into a clinical difference in infection rate is unclear (the standard procedure is to sterilize to 10^−6^ for operative devices) [37,38]. Supporters of Alloderm-RTU advocate based on its long term data on safety and effectiveness, although most studies were performed with the original freeze-dried version (sterilized to 10^−6^) and not the RTU product [3,9,10,35,36].

The primary outcome of the study was seroma formation and drain duration. Seromas following prosthetic based breast reconstruction are common and can be associated with infection and prosthetic loss due to conditions created such as a hypovascular, proinflammatory milieu of the mastectomy skin flap, a geometrically complex dead space, and the presence of a foreign body with potential contamination and biofilm [17,18,19,20]. Higher drainage volume also leads to a longer duration of drains and disrupts the postoperative life of the patient [20,21]. The use of a drain itself poses the risk of open communication with skin and external flora, allowing for direct colonization of the wound site and greatly increased the risk of wound infection and possible failure [19,21]. Although a trend existed for a higher drain duration with Alloderm-RTU, our results do not show a statistically significant or clinically meaningful difference in these outcomes between the two groups. In addition, the overall rates of minor complications were statistically similar between the two groups; although Alloderm-RTU had 3 times as much wound infection requiring antibiotics as DermACELL. The incidence of major complications (unplanned return to the operating room, loss of implant) was also not statistically significant between the two groups, although patients in the Alloderm-RTU group had twice as many unplanned return trips to the operating room as DermACELL.

It should be noted that our results (drain duration and incidence of minor and major postoperative complications) are either similar or lower than reported in previously published studies. The majority of these studies are retrospective [37,38,39,40,41], although three of these studies did have direct comparisons of Alloderm-RTU and Dermacell [37,38,39,40]. Zenn et al. performed a retrospective study of 140 breasts including both immediate and delayed reconstruction and showed no statistically significant difference between Alloderm-RTU and DermACELL in infections (0.8% vs. 1.7% respectively, *p* = 0.75) or revisional surgery (5.4% vs. 4.2% respectively, *p* = 0.57) [37]. In contrast, another retrospective study of 100 breasts including both tissue expander and direct-to-implant reconstruction showed a longer time to drain removal with Alloderm-RTU compared to DermACELL (20.6 days vs. 15.8 days, *p* = 0.017) and higher incidence of red breast syndrome (26% vs. 0%, *p* = 0.0001), with similar rates for loss of implant and cellulitis [38]. A recent retrospective chart review of 64 patients with both tissue expander and direct-to-implant reconstruction by Greig et al. showed no difference in capsular contraction, implant replacement, complication rates of seroma, hematoma, mastectomy flap necrosis, and infection [39].

There are only two prospective randomized controlled trials involving ADMs in direct-to-implant subpectoral reconstruction [41,42]. Both involve Alloderm-RTU but none involve DermACELL. The first study compared Alloderm-RTU with Allomax^TM^ (Bard Davol Inc, Cranston, RI, USA). This study focused on histologic findings and may have been underpowered as there were 15 patients in each arm but complication rates of 8% (Alloderm-RTU) versus 26% (AlloMax^TM^, C.R. Bard, Warwick, RI, USA) were not found to be statistically significant [41]. The second study compared Alloderm-RTU to another human-derived ADM called Cortiva 1 mm (RTI Surgical, Alachua, FL, USA) in a prospective randomized trial [42]. Although the trial is still ongoing, an interim analysis of 59 breasts in the subpectoral study arm revealed no statistically significant difference in terms of postoperative outcomes including drain duration of 17 days in both arms.

The limitations of the current study include the relatively small sample size, the conduct of the study at a single centre, and its open-label design. However, this is the only prospective trial we are aware of comparing the two most used ADMs in North America (Alloderm-RTU and DermACELL) in a head-to-head randomized prospective study, and thus it represents novel and valuable information. Clearly, the plastic surgeons could not be blinded to the ADM type once the randomization occurred at the patient level; so conceivably this could have played a role in the difference in outcomes between the two types of ADMs. Even though three plastic surgeons were performing the procedures, the majority of the cases (2/3) were done by one plastic surgeon and thus accounting for possible differences in surgical technique by the plastic surgeon was not possible with the small sample size. It should be noted that the majority of the drains were removed by the outpatient home care nurse, which did not know the type of ADM used. There was also an imbalance in the median mastectomy weights in the two study arms, while this could in part be related to missing data that could not be found in the medical records, the difference was not statistically significant.

While a formal economic analysis would have been useful for this study, this was not prospectively planned. Economic analysis requires the incorporation of appropriate economic tools to be used throughout the study. Such an analysis would have also been challenging in the current study as at the time of our study, the vendors for the two ADM companies were in the process of competing for a Request for Proposal (RFP) at our institution, and as such the prices of the products were in flux. These fluctuating prices would also impair our ability to perform a robust economic analysis for the study. Alloderm-RTU and DermACELL are considered to be amongst the more popular but also more expensive ADM products in the breast reconstruction market. Alternative synthetic meshes such as those used for hernia repairs are also being used in breast reconstruction and are relatively less expensive [22,43]. The purpose of the study was to evaluate the two most commonly used ADM’s. However, given the frequency of immediate breast reconstructive surgery, further trials are needed comparing important endpoints such as; patient satisfaction, hospital admission, delays in adjuvant breast cancer therapy, and health economics in this era of value-based care. 

## 5. Conclusions

The current trial compared two commonly used ADMs in a head-to-head trial. There was no statistically significant difference in drain duration, minor or major complications between DermACELL over Alloderm-RTU in immediate subpectoral permanent implant-based breast reconstruction post-mastectomy. Future studies should assess the cost of using each type of ADM which ideally should include not only the price of the product but also the healthcare costs of managing unplanned complications and reconstructive failures.

## Figures and Tables

**Table 1:** Baseline demographic and clinical characteristics. All values are number (percentage) unless otherwise specified.

Baseline Characteristic	All	Alloderm-RTU	DermACELL
Number of Breasts (%)	81	41 (50.6)	40 (49.4)
Number of Patients (%)	62	33 (50.0)	33 (50.0)
Age, years, mean (SD)	49.6 (11.1)	47.8 (11.1)	51.4 (11.0)
BMI, kg/m^2^, mean (SD)	24.9 (4.7)	24.9 (4.6)	24.9 (4.9)
Smoking history			
Current (%)	4 (6.5)	3 (9.7)	1 (3.2)
Prior (≥1 month before) (%)	19 (30.7)	9 (29.0)	10 (32.3)
Never (%)	39 (62.9)	19 (61.3)	20 (64.5)
Diabetes (%)	1 (1.6)	0 (0)	1 (3.2)
Heart disease ^a^ (%)	8 (12.9)	5 (16.1)	3 (9.7)
Preoperative chemotherapy	7 (11.3)	4 (12.9)	3 (9.7)
Prior radiotherapy (%)	13 (16.1)	5 (12.2)	8 (20.0)
Breast size D cup or greater (%)	11 (17.7)	4 (12.9)	7 (22.6)
Ptosis grade III or greater (%)	4 (4.9)	0 (0)	4 (10.0)

^a^ Includes hypertension, CCF, Stroke, Angina, CAD.

**Table 2:** Surgical characteristics. All values are number (percentage) unless otherwise indicated.

Surgical Characteristic	All *n* = 78	Alloderm-RTU *n* = 38	DermACELL *n* = 40
Therapeutic indication for surgery ^a^	53 (67.9)	25 (65.8)	28 (70.0)
Mastectomy Type			
Nipple sparing	40 (51.3)	21 (55.3)	19 (47.5)
Inframammary incision	34	18	16
Incision without vertical	2	0	2
Vertical or diagonal	5	3	1
Skin sparing	38 (48.7)	17 (44.7)	21 (52.5)
Incision without vertical component	13	5	8
Vertical or diagonal	21	12	9
Wise pattern	4	0	4
Axillary surgery performed	52 (66.7)	25 (65.8)	27 (67.5)
Sentinel node biopsy ^b^	49	23	26
Mastectomy weight, g, median (IQR)	467 (276, 665)	600 (324, 714)	387 (269, 542)

^a^ compared to prophylactic indication, ^b^ compared to axillary lymph node dissection.

**Table 3:** Clinical endpoint data. All values are number (percentage) unless otherwise indicated.

Clinical Outcomes	All *n* = 78	Alloderm-RTU *n* = 38	DermA-CELL *n* = 40	*p*-Value		Risk Difference (95% CI)
				1 *	2 *	
Mean duration of drain (SD), days	10.0 (5.0)	10.8 (5.5)	9.2 (4.5)	0.16	0.15	1.6 (−0.7 to 3.9)
**Minor complications**						
Seromas requiring aspiration post drain removal	7 (9.0)	2 (5.3)	5 (12.5)	0.43	0.28	−7.2 (−19.7 to 5.2)
Red breast syndrome	2 (2.6)	1 (2.6)	1 (2.5)	1.00	0.97	0.1 (−6.9 to 7.2)
Wound dehiscence	5 (6.4)	3 (7.9)	2 (5.0)	0.67	0.68	2.9 (−8.0 to 13.8)
Requiring return to OR	2	1	1			
Wound infection requiring antibiotics	4 (5.1)	3 (7.9)	1 (2.5)	0.35	0.32	5.4 (−4.5 to 15.2)
Requiring return to OR	3	2	1			
Hematoma	2 (2.6)	2 (5.3)	0 (0.0)	0.23	-	5.3 (−1.8 to 12.4)
Requiring return to OR	1	1	0			
Skin necrosis	6 (7.7)	2 (5.3)	4 (10.0)	0.68	0.77	−4.7 (−16.4 to 7.0)
Requiring return to OR	3	2	1			
Capsular contracture	1 (1.3)	1 (2.6)	0 (0.0)	0.49	-	2.6 (−2.5 to 7.7)
**Major complications**						
Return to OR	9 (11.5)	6 (15.8)	3 (7.5)	0.30	0.28	8.3 (−5.9 to 22.5)
Suture removal	1	1	0			
Wound dehiscence	2	1	1			
Infection	3	2	1			
Hematoma	1	1	0			
Skin necrosis	3	2	1			
Removal of implant	4	2	2			
Loss of Implant	4 (5.1)	2 (5.3)	2 (5.0)	1.00	0.96	0.3 (−9.5 to 10.1)

* 1 = assumes no correlation between breasts; 2 = assumes correlation between breasts.

**Table 4:** Post-surgical treatment and follow up. All values are number (percentage) unless otherwise indicated.

Post-Surgical Outcome	All	Alloderm-RTU	DermACELL	*p*-Value	
				1 *	2 *
*n* Patients Evaluable	59	28	31		
Final evaluation by plastic surgeon ^a^	49 (83.1)	22 (78.6)	27 (87.1)	0.49	-
Median number of days between surgery and first plastics assessment (IQR)	7 (6, 9)	7 (6, 9)	7 (6, 9)	0.81	-
Median number of weeks between surgery and final assessment (IQR)	9 (5, 17)	13.5 (6, 19)	8 (4, 14)	0.10	-
*n* Breasts Evaluable	78	38	40		
Median number of plastic surgeon visits (IQR)	4 (3, 5)	4 (3, 5)	3 (3, 4.5)	0.13	0.80
Indication for adjuvant radiotherapy	13 (16.7)	7 (18.4)	6 (15.0)	0.77	0.66
Indication for adjuvant chemotherapy	16 (27.1)	9 (32.1)	7 (22.6)	0.56	0.41

* 1 = assumes no correlation between breasts; 2 = assumes a correlation between breasts, ^a^ compared to evaluation by breast surgeon.
